# Effect of nutritional condition on photosymbiotic consortium of cultured *Globigerinoides sacculifer* (Rhizaria, Foraminifera)

**DOI:** 10.1007/s13199-017-0530-3

**Published:** 2017-12-09

**Authors:** Haruka Takagi, Katsunori Kimoto, Tetsuichi Fujiki, Kazuyoshi Moriya

**Affiliations:** 1 Graduate School of Creative Science and Engineering, Waseda University, 1-6-1 Nishiwaseda, Shinjuku, Tokyo, 169-8050 Japan; 2 Present Address: Atmosphere and Ocean Research Institute, The University of Tokyo, 5-1-5 Kashiwanoha, Kashiwa, Chiba, 277-8564 Japan; 3 Research and Development Center for Global Change, Japan Agency for Marine-Earth Science and Technology, 2-15 Natsushima-cho, Yokosuka, Kanagawa 237-0061 Japan; 4 Department of Earth Sciences, Faculty of Education and Integrated Arts and Sciences, Waseda University, 1-6-1 Nishiwaseda, Shinjuku, Tokyo, 169-8050 Japan

**Keywords:** Planktic foraminifers, Photosymbiosis, Symbiotic algae, Nutrition, Growth, Photophysiology

## Abstract

Several foraminifers found in warm and low-nutrient ocean surface water have photosynthetic algae as endosymbionts (photosymbiosis). To understand the trophic interactions, we studied *Globigerinoides sacculifer*, a spinose planktic foraminifer that has a dinoflagellate endosymbiont. We controlled two nutritional factors, feeding and inorganic nutrients in the seawater. The growth of the host and the symbionts and the photophysiological parameters were monitored under four experimental conditions. The results demonstrated that the holobionts primarily relied on phagotrophy for growth. The foraminifers grew considerably, and the chlorophyll *a* content per foraminifer, which is an indicator of the symbiont population, increased in the fed groups, but not in the unfed groups. The nutrient-rich seawater used for some of the cultures made no difference in either the growth or photophysiology of the holobionts. These observations indicated that the symbionts mainly utilized metabolites from the hosts for photosynthesis rather than inorganic nutrients in the seawater. Additionally, we observed that the symbionts in the starved hosts maintained their photosynthetic capability for at least 12 days, and that the hosts maintained at least some symbionts until gametogenesis was achieved. This suggests that the hosts have to retain the symbionts as an energy source for reproduction. The symbionts may also play an indispensable role in the metabolic activities of the hosts including waste transport or essential compound synthesis. Overall, our results revealed a novel mode of photosymbiosis in planktic foraminifers which contrasts with that found in benthic photosymbiotic foraminifers and corals.

**Electronic supplementary material:**

The online version of this article (10.1007/s13199-017-0530-3) contains supplementary material, which is available to authorized users.

## Introduction

**Fig. 1:**
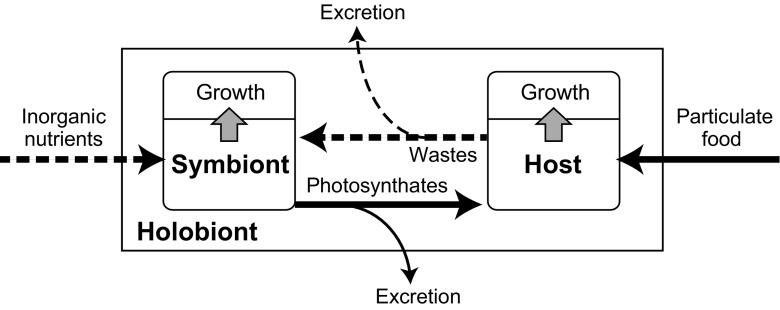
Schematic representation of the possible trophic interactions in the photosymbiotic foraminifer-algal consortium (holobiont). Solid arrows represent the flow of nutrients in organic forms. Dashed arrows represent the flow of nutrients in dissolved inorganic forms

Algal photosymbiosis is a form of mixotrophy (hybrid mode of nutrition with both phagotrophy and phototrophy) (Stoecker 1998). It allows for greater flexibility regarding resource acquisition as dissolved inorganic nutrients are obtained by the algal symbionts, and organic particulate foods are made available through feeding by the host (Fig. 1). This pattern is commonly observed in various marine organisms such as hermatypic corals, sea anemones, foraminifers, and radiolarians, especially those inhabiting oligotrophic environments (e.g. Muscatine 1971; Anderson et al. 1983; Muscatine et al. 1984; Lee 1998; Caron 2000). Recently, metagenomic studies and in situ estimations of marine plankton biomass have shown that photosymbiotic protists play an important role in food webs and biogeochemical cycles in oligotrophic part of oceans (de Vargas et al. 2015; Biard et al. 2016). However, despite this, photosymbiosis in plankton are poorly understood at the organismal level, compared with that in benthic organisms. This is partly because of difficulties in culturing such minute free-floating plankton.

Planktic foraminifers are protistan zooplankton that prey on other plankton including copepods, ciliates, and microalgae (Anderson et al. 1979; Spindler et al. 1984; Hemleben et al. 1989). To date, approximately ten species of planktic foraminifers have been recognized to be photosymbiotic with eukaryotic algae (Gastrich 1987; Hemleben et al. 1989). Furthermore, a cyanobacterial symbiosis was recently reported for one species, *Globigerina bulloides* (type IId) (Bird et al. 2017). Studies on planktic foraminifers have shown that photosymbiotic species are characteristic of tropical to subtropical surface-water masses, which are usually nutrient-limited (Murray 1897; Bé 1977). This suggests that photosymbiosis is a successful ecological strategy for planktic foraminifers that live in oligotrophic water as photosynthates by the symbionts could serve as an important nutritional source for the host (e.g. Caron 2000; Yellowlees et al. 2008).

Interaction between hosts and symbionts has been investigated thoroughly in reef-dwelling benthic organisms, such as hermatypic corals and larger benthic foraminifers. In nutrient-limited reef environments, since the host controls the nutrient supply to the symbionts, photosynthates produced by the symbionts primarily comprise carbohydrates and lipids (Muscatine 1967). These compounds can support their respiration, but not the synthesis of proteins or nucleic acids, required for growth and reproduction. Therefore, the symbiont population is regulated to an almost constant density, when the host corals are in a healthy condition (Falkowski et al. 1993; Dubinsky and Jokiel 1994). The dissolved inorganic nutrients in seawater affect the physiology of such host-symbiont systems (Lee et al. 1991; Yellowlees et al. 2008; Tanaka et al. 2014; Rosset et al. 2015). When nutrients are abundant in the seawater, it promotes the proliferation of the symbionts. Under these conditions, the reef-dwelling larger benthic foraminifer, *Heterostegina depressa*, that is symbiotic with diatoms is reported to be capable of normal growth without external food sources (Röttger and Berger 1972). This implies that the photosynthates from the symbionts nourish their host. However, excess nutrients can cause the collapse of the photosymbiosis as the symbionts utilize the photosynthates for their own growth and reduce the supply to their hosts, which lose the control of the symbiont population (Falkowski et al. 1993; Lee 1998). In these benthic taxa, inorganic nutrients outside the membrane of the host appear to be available to the symbionts.

Studies on photosynthesis in symbiont-bearing planktic foraminifers have examined oxygen generation, carbon fixation, chlorophyll levels, and photophysiology (e.g. Jørgensen et al. 1985; Spero and Parker 1985; Rink et al. 1998; Lombard et al. 2009; Fujiki et al. 2014; Takagi et al. 2016). An early experimental study showed that the rates of oxygen generation through photosynthesis in symbionts of *Globigerinoides sacculifer* greatly exceeded the holobiont respiration rates (Jørgensen et al. 1985). Based on the oxygen budget, potential photosynthetic rates theoretically account for the entire carbon requirement of the host foraminifer for its metabolism and growth (Jørgensen et al. 1985; Lombard et al. 2009). However, another study on *G. sacculifer* with numerous photosynthesizing symbionts, showed that it was unable to grow without phagotrophy, and died prematurely (Bé et al. 1981; Caron et al. 1981). These findings indicated that photosynthates produced by the symbionts were insufficient for sustaining the growth of the host. Furthermore, it is pointed out that the diffusion-limited supply of nitrogen and phosphorus to symbionts within the cytoplasm of planktic foraminifers was not sufficient to support optimal photosynthetic rates in the symbiont (Jørgensen et al. 1985; Spero et al. 1991; Zeebe et al. 1999). As a result, feeding by foraminifers was required (Jørgensen et al. 1985; Uhle et al. 1999). However, none of the studies have examined the effect of elevated nutrient concentration in seawater on photosymbiotic planktic foraminifers.

When there is a sufficient supply of inorganic nitrogen and phosphorus, photosynthates of the symbionts may facilitate the growth of the host foraminifers without a food supply. Alternatively, based on what is known about inorganic nutrients and benthic organisms, the photosymbiotic system may collapse following an explosion in the symbiont population. To examine this, one approach is to investigate photosymbiotic relationships using active chlorophyll fluorometry. Chlorophyll fluorescence can serve as a proxy for various evaluations of photosynthesis, specifically photosystem II (PSII) chemistry. The results can be used as an indicator of the health of the photosymbiotic systems (e.g. Roth 2014). For example, the parameter *F*_v_/*F*_m_ (maximum quantum yield of PSII chemistry) has been widely used as a diagnostic tool to analyze nutrient stress in phytoplankton (Kolber et al. 1988; Geider et al. 1993). Generally, high *F*_v_/*F*_m_ values indicate good conditions for the phototrophs, although the robustness of the measure depends on the growth condition of algae, and whether it is unbalanced or balanced growth (Parkhill et al. 2001). If the latter, *F*_v_/*F*_m_ may be almost independent of nutrient limitation; thus interpretations should be made carefully (Parkhill et al. 2001; Suggett et al. 2009). Other photophysiological parameters like the functional absorption cross-section of PSII (σ_PSII_) and time constant of initial electron acceptor Q_A_ re-oxidization (τ_QA_) are helpful in assessing the effect of nutritional conditions on the photophysiological system of holobionts. σ_PSII_ represents the efficiency of energy transfer from antenna pigments to PSII reaction centers (RCII). The composition of accessory photopigments and amount of pigments relative to RCII can affect the σ_PSII_ value (Kolber et al. 1988). τ_QA_ represents the minimum turnover time for electron transport, and is governed by the rate of the downstream electron transport. It is also affected by the ratio of RCII to carbon fixation capacity, which can be changed as a photoacclimation response (Sukenik et al. 1987; Moore et al. 2006). Active chlorophyll fluorometry can thus provide an understanding of the photochemical activity of PSII over time in a noninvasive manner and assess the physiological state of the symbionts, and thereby, the host foraminifers.

The organism used in the present study was *Globigerinoides sacculifer*. This is a spinose planktic foraminifer that has a dinoflagellate-endosymbiont. This species spreads out its numerous symbionts along the spines during the light period, forming a concentric spherical halo surrounding the test (Anderson and Bé 1976). It is one of the best-studied planktic foraminifers in laboratory culture for investigating growth, calcification, longevity, feeding, and photosymbiosis (Bé et al. 1981, 1982, 1983; Caron et al. 1981). Moreover, based on the ribosomal DNA regions SSU and ITS-1, *G. sacculifer* is revealed to comprise only a single genotype (André et al. 2013), ensuring that our study was free from potential variations caused by genetic differences at cryptic species level. *Globigerinoides sacculifer* harbors only one symbiont species, *Pelagodinium béii* (Spero 1987; Shaked and de Vargas 2006; Siano et al. 2010). This alga is known to comprise four genetic subgroups, based on the LSU and ITS-2 regions of ribosomal DNA (Shaked and de Vargas 2006). Other dinoflagellate-bearing planktic foraminifers, *Globigerinoides conglobatus*, *Globigerinoides ruber*, and *Orbulina universa*, as well as radiolarians *Acanthochiasma* spp. are reported to be in symbiosis with this algal species (Gast and Caron 2001; Shaked and de Vargas 2006; Decelle et al. 2012). *Pelagodinium* is a sister group to the genus *Symbiodinium*, the well-known symbionts of corals and benthic foraminifers (Shaked and de Vargas 2006).

The aim of the present study was to evaluate the effect of nutritional condition on the *Globigerinoides sacculifer* photosymbiotic consortium, with particular reference to the growth of both the host and symbionts, as well as their photophysiology. It was anticipated that our study on planktic foraminifers would provide new perspectives on photosymbiosis in plankton, which are important in pelagic ecosystems.

## Materials and methods

### Foraminifer samples

The sampling and nutrient-controlled culture experiment were conducted at Sesoko Station, Tropical Biosphere Research Center, University of the Ryukyus in Japan, over the same period as the work by Takagi et al. (2016). Specimens were collected from the East China Sea offshore of Sesoko Island, Okinawa, Japan (26°37.3′N, 127°52.3′E, 60-m deep) on November 29th, 2013. A plankton net (63-μm mesh, 45-cm aperture) was towed in the near-surface water (<15 m). The surface-water temperature, salinity, and chlorophyll *a* (Chl *a*) concentration in the sampling site were 23.7 °C, 34.6, and 0.3 μg L^−1^, respectively. In the laboratory, live *G. sacculifer* were sorted and isolated using Pasteur pipettes under a dissecting microscope.

### Experimental setup and culture protocols

To examine the effect of abundant inorganic nutrients and particulate food, four experimental groups were established— (a) group SWf; fed every other day and cultured in low-nutrient seawater, (b) group SW; unfed, cultured in low-nutrient seawater, (c) group NPf; fed every other day, cultured in high-nutrient seawater, and (d) group NP; unfed, cultured in high-nutrient seawater (Table 1). *Artemia salina* nauplii were used for feeding. A feeding rate of one *Artemia* nauplius in two days was used in this study and is in the range for carnivorous planktic foraminifers (daily feeding, Spindler et al. 1984; one feeding event in 3.3 days, Caron and Bé 1984). Specimens fed at this feeding rate have been shown to grow well in laboratory cultures (Bé et al. 1981; Spero and Lea 1993; Lombard et al. 2009). The phosphorus content (total organic phosphorus + orthophosphate) of an *Artemia* nauplius is reported to be approximately 14–27 ng *Artemia* ^−1^ (= 0.45–0.87 nmol *Artemia* ^−1^) (Wijgerde et al. 2011). Therefore, assuming that 100% of phosphorus in a single *Artemia* individual is remineralized and supplied to the symbionts, phosphorus flux was calculated as 0.018 nmol h^−1^ on average (= 0.87 nmol *Artemia* ^−1^ 48 h^−1^).

**Table 1:** Culture conditions for the four experimental groups. The organism fed was *Artemia salina* nauplius (see text for detail)

Experimental group	Culture medium			Feeding *Artemia*
	Seawater treatment	NO_3_ + NO_2_ (μmol L^−1^)	PO_4_ (μmol L^−1^)	
SWf	0.22 μm-filtration	0.2	0.07	1 every 2 days
SW	0.22 μm-filtration	0.2	0.07	Unfed
NPf	0.22 μm-filtration, nutrients added	16	1	1 every 2 days
NP	0.22 μm-filtration, nutrients added	16	1	Unfed

The nutrient concentration for the high-nutrient seawater groups was set to supply phosphorus at an amount comparable with that of remineralized phosphorus from an *Artemia* individual as calculated above. In a diffusion-limited environment, the diffusion flux of nutrients in the layer surrounding a foraminifer called the symbiont halo is given by

$$ Nutrient\kern0.17em flux=4\pi DRS $$

where *D* is the molecular diffusion coefficient for the nutrient, *R* is the radius of the hypothetical sphere of photosynthesis, and *S* is the nutrient concentration in the culture medium (Jørgensen et al. 1985). Herein, we considered *D* as 2.69 mm^2^ h^−1^ (molecular diffusion coefficient for HPO_4_^2−^ at 27 °C, Boudreau 1997), *R* as 0.5 mm for specimens with a test size of ca. 400 μm (assuming symbiont halo width to be 300 μm, Jørgensen et al. 1985; Uhle et al. 1999). To achieve a phosphorus flux of 0.018 nmol h^−1^, concentration *S* was calculated as 1.06 μmol L^−1^. Based on this estimation, the phosphorus concentration in the seawater for the high-nutrient groups was set as 1 μmol L^−1^. The nitrogen concentration was set as 16 μmol L^−1^ which is supposed as sufficient to achieve a balanced growth of the symbionts at the above mentioned phosphorus concentration (N:*P* = 16:1). The nitrogen and phosphorus concentrations were adjusted by adding sodium nitrate (NaNO_3_) and sodium dihydrogen phosphate (NaH_2_PO_4_·2H_2_O) to 0.22 μm-filtered seawater collected at the sampling site. The concentrations of nitrogen (NO_3_ + NO_2_) and phosphorus (PO_4_) in the originally collected seawater were 0.23 and 0.07 μmol L^−1^, respectively (Table 1). The filtered seawater served as the culture medium for the low-nutrient groups. The conditions for the SWf group simulated those in the natural environment of foraminifers. Some on this group were reported by Takagi et al. (2016).

The experiment was initiated with 18 *G. sacculifer* specimens in each experimental group. Considering the short longevity of planktic foraminifers (ca. one lunar month, Hemleben et al. 1989), the age in days of individuals at the beginning of the experiment could be an important factor in culture experiments. In addition, time since the last feeding and the time since the last chamber formation could affect the growth profiles. It is ideal if cloned individuals with a fixed period of acclimation to the culture conditions can be used. This is the usual strategy for experiments on benthic foraminifers and corals (e.g. Hikami et al. 2011; Hayashi et al. 2013). Unfortunately, cloned individuals are not available for planktic foraminifers. Furthermore, foraminifers grow rapidly and controling conditions prior to the experiment is not practical. Thus experiments in this study involve some uncertainties. The specimens used were screened based on measured initial conditions, i.e., test size, Chl *a* content, and photophysiological parameters. At the beginning of the experiments, these parameters applied to every group and there were no statistical differences (Table 2), indicating that each experimental group, from this aspect was identical.

**Table 2:** Initial conditions for each group and the results of a one-way analysis of variance (one-way ANOVA) for each parameter. The initial conditions did not differ among the groups. The mean and error (1σ) of each of 18 specimens in each group are shown. Chl *a*; chlorophyll *a*, *F*_v_/*F*_m_; maximum quantum yield of photosystem II chemistry, σ_PSII_; functional absorption cross-section of photosystem II, τ_QA_; time constant of initial electron acceptor Q_A_ re-oxidization, *F*; statistical index (*F*-value) with degree of freedom in subscripts, *p*; *p*-value

	Test size (μm)	Chl *a* (ng foraminifer^−1^)	*F*_v_/*F*_m_	σ_PSII_ (× 10^−20^ quanta^−1^)	τ_QA_ (μs)
SWf	382 ± 129	44 ± 35	0.485 ± 0.021	666 ± 68	416 ± 59
SW	371 ± 121	33 ± 29	0.485 ± 0.025	609 ± 73	461 ± 87
NPf	411 ± 112	47 ± 46	0.492 ± 0.018	648 ± 76	421 ± 79
NP	432 ± 123	33 ± 36	0.484 ± 0.031	612 ± 86	444 ± 68
One-way ANOVA	*F*_1,70_ = 2.24 *p* = 0.13	*F*_1,70_ = 0.22 *p* = 0.64	*F*_1,70_ = 0.02 *p* = 0.88	*F*_1,70_ = 2.27 *p* = 0.13	*F*_1,70_ = 0.30 *p* = 0.59

The specimens were maintained in culture dishes (Nunclon 6-well Multidishes, Nunc International) filled with the respective culture media. Each individual was placed in a single well (17 mL). The culture dishes were maintained in a water bath at 27 ± 0.5 °C. Almost all of the seawater in each well was replaced daily with a new aliquot to maintain the characteristics of the seawater as constant as possible. Irradiance (photosynthetically active radiation, wavelength of 400–700 nm) was controlled at 200 ± 30 μmol photons m^−2^ s^−1^ (cf. Jørgensen et al. 1985; Rink et al. 1998) using metal halide lamps (Funnel 2, Kamihata Fish Industries Ltd.) set above the water bath to achieve saturation of photosynthesis. The irradiance was determined using a quantum sensor (LI-190SA, Li-Cor). A 14/10 h light/dark cycle was used in this study. Fast repetition rate (FRR) fluorometric measurements (see below) were conducted for each specimen during the culture period. After the measurement, each specimen was photomicrographed, and the test size was measured using a dissecting microscope with a calibrated eyepiece. Each experiment was conducted for 14 days.

### FRR fluorometric measurement for holobionts

The protocol of the FRR fluorometric measurement followed in this study was described in detail by Fujiki et al. (2014) and Takagi et al. (2016). The fluorometric measurements were performed during daytime. A FRR fluorometer (Diving Flash, Kimoto Electric Co., Ltd.; for the details of the instrument, see Fujiki et al. 2008) was used to assess the photophysiological conditions and Chl *a* content of the symbiotic algae within the foraminifers.

The fluorescence induction curve of PSII was obtained from FRR fluorometry. The fluorescence induction curve was numerically fitted by using the procedure described by Kolber et al. (1998), and PSII parameters were calculated. The parameters analyzed in this study were minimum fluorescence (*F*_0_), maximum fluorescence (*F*_m_), variable fluorescence (*F*_v_), potential photochemical efficiency (*F*_v_/*F*_m_), functional absorption cross-section of PSII (σ_PSII_), and time constant of initial electron acceptor Q_A_ re-oxidization (τ_QA_). The Chl *a* content in an individual foraminifer could be estimated from its *F*_m_ value based on the linear relationship between them (Fujiki et al. 2014). In this study, we calculated the Chl *a* content in each foraminifer specimen with the linear function previously established (Takagi et al. 2016).

### FRR fluorometric measurement on free-living symbionts in culture

To compare the photophysiology of the symbiotic dinoflagellate species *Pelagodinium béii* in its host with algae that are free-living under nutrient-replete conditions, cultures of *P. béii* were evaluated by the FRR fluorometry. The *P. béii* culture (NIES-4008, GenBank accession number LC333575) was originally isolated from the host foraminifer *G. sacculifer* collected in the Northwestern Pacific Ocean during a sampling cruise (R/V Mirai operated by the Japan Agency for Marine-Earth Science and Technology; cruise No. MR13–04). It was isolated onboard, and has been maintained at the Microbial Culture Collection at the National Institute for Environmental Studies (NIES, Tsukuba, Japan). The culture was maintained at 21 °C in white fluorescent light (170 μmol photons m^−2^ s^−1^) with a 12/12 h light/dark cycle in nutrient-replete medium (ESM medium, 25 mL). The medium contained 120 mg of NaNO_3_, 5 mg of K_2_HPO_4_, 0.001 mg of vitamin B_12_, 0.001 mg of biotin, 0.1 mg of thiamin-HC1,0.259 mg of Fe-EDTA, 0.332 mg of Mn-EDTA, 1 g of Tris (hydroxymethyl) aminomethane, 25 mL of soil extracts, and 975 mL of seawater in one liter (Okaichi et al. 1982). Two sequential generations of the culture were utilized: one in the exponential growth phase (7 days after subculture), and the other in the saturation phase (10 days after subculture). The growth profiles of the cultures were monitored daily by assessing the relative intensity of chlorophyll fluorescence using a fluorometer (FluorPen FP100, Photon Systems Instruments Ltd.). Photophysiological parameters were obtained using the same FRR fluorometric equipment as that used for the holobiont measurements.

### Data analysis

Nonlinear mixed models were employed in this study within a Bayesian modelling framework using Markov chain Monte Carlo (MCMC) simulation to understand the photophysiological response through time for each group. Individual ID was used as a random factor in the models. The models were examined by means of the Bayesian modelling package rstanarm (Stan Development Team 2016) in R (R version 3.3.1, R Core Team 2016). Each model was run with four chains for 1000 warm-up and 1000 sampling steps. For all parameters in the models, the convergence measure $\widehat{R}$ was <1.005 ($\widehat{R}$less than 1.1 indicates adequate convergence, Gelman et al. 2003). The posterior predictive distribution and its 95% interval were estimated from the established model for each parameter.

To compare the photophysiological difference among the experimental groups, the difference (Δ) in each parameter of the predictive MCMC samples between two groups was simulated. The effect of feeding was assessed by comparing the photophysiological parameters between SWf and SW (Δ_SWf − SW_), and between NPf and NP (Δ_NPf − NP_). As such, the differences between NPf and SWf (Δ_NPf − SWf_), and NP and SW (Δ_NP − SW_) were simulated to assess the effect of inorganic nutrients. The 95% predictive interval of the posterior distribution was used to evaluate the significance of the effect. In this study, when the 95% posterior predictive interval of a difference (Δ) contained 0, it implied that the difference between the two groups was not significant at 95% probability.

## Results

### Growth of foraminifers

**Fig. 2:**
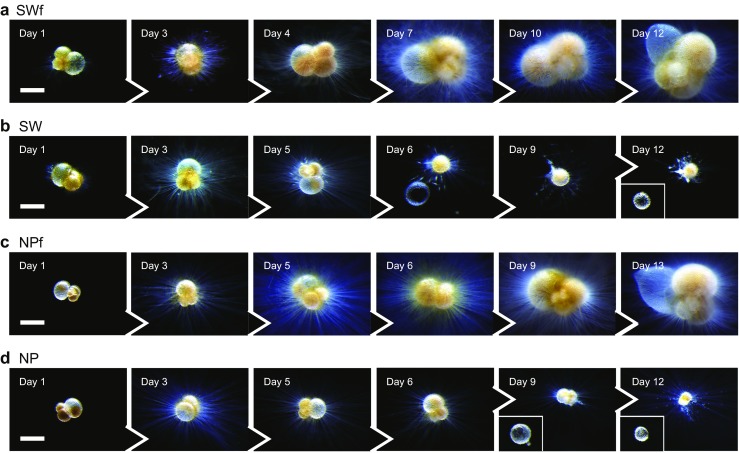
Time-series light micrographs of selected specimens during the experiment. **a** Group SWf, specimen sac18, **b** Group SW, specimen sac25, **c** Group NPf, specimen sac68, and **d** Group NP, specimen sac69. The shedding of chamber(s) was often observed in the unfed specimens in groups SW and NP (b, d). Scale bars represent 200 μm

**Fig. 3:**
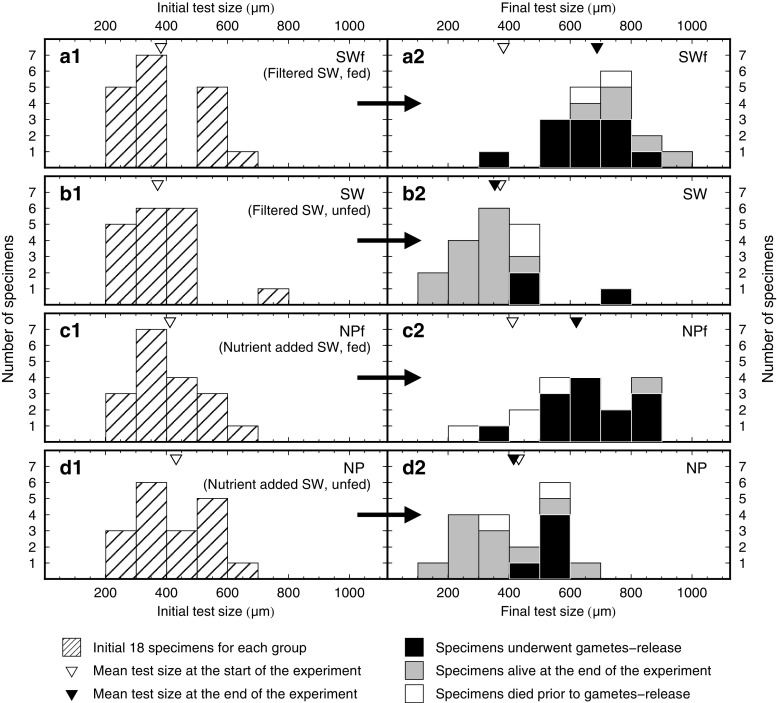
Histograms of initial and final test sizes in the 4 experimental groups. **a** Group SWf, **b** Group SW, **c** Group NPf, and **d** Group NP. Shadings in the final condition (a2, b2, c2, and d2) indicates the reproductive state of the specimens at the end of the experiment. Open and filled triangles represent the mean initial and final test sizes, respectively

**Fig. 4:**
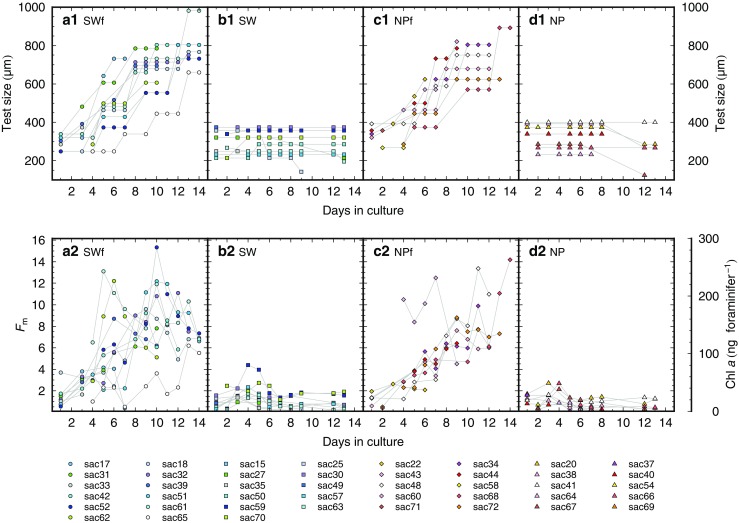
Longitudinal changes in the test size (a1 to d1) and the *F*_m_ value (Chl *a* content) (a2 to d2). **a** Group SWf, **b** Group SW, **c** Group NPf, and **d** Group NP. The decrease in test size indicates that the specimen shed their chamber(s) spontaneously

There was a clear difference between the growth profiles of the fed and unfed groups (Figs. 2 and 3). The final mean test size was larger in the fed groups (SWf, 688 μm; NPf, 621 μm) than that in the unfed groups (SW, 353 μm; NP, 415 μm). In the fed groups, new chambers were formed once in 3–4 days in most cases (Fig. 4), forming tests in normal trochospire (Fig. 2). The maximum number of chambers formed for a given individual was three. The majority of the grown specimens formed a sac-like ultimate chamber. Test size increased by +306 μm (SWf) and +210 μm (NPf) in group means. In contrast, growth in the unfed groups was significantly suppressed. Chamber formation, if any, was only observed by day 5 (e.g. sac70 in SW, Fig. 4 b1), and it was not observed from day 6 to the end of the experiment. At most, only one chamber per individual was formed. Some specimens in the unfed groups shed their original or newly precipitated chamber(s) (Fig. 2c, d), which was not observed in the fed groups. This resulted in the test sizes being smaller at the end of the experiment than at the initial stages.

The findings on reproduction are summarized in Fig. 3. The numbers of specimens that released gametes were 11 (SWf), 3 (SW), 13 (NPf), and 5 (NP). Since total mortality including death of matured hosts (after gamete release and subsequent natural death) was low in the unfed groups, the number of specimens alive on the final day of the experiment was higher in the unfed groups (SW, 13; NP, 11) than in the fed groups (SWf, 5; NPf, 1). In the fed groups, the specimens with initial test sizes greater than 400 μm reached reproductive maturation so soon that time-series data could not be collected. Therefore, subsequently we used the data from specimens with an initial test size smaller than 400 μm. This enabled us to analyze the longitudinal trend of the Chl *a* content and photophysiological parameters.

### Chlorophyll *a* content

Overall, the Chl *a* content per foraminifer increased in the fed groups (Fig. 4). The maximum Chl *a* content reached 281 ng foraminifer^−1^ in SWf (sac52, day 10, 554 μm) and 260 ng foraminifer^−1^ in NPf (sac68, day 14, 893 μm). These values were more than 5 times higher than those determined initially for the specimens. Both the intra-specimen fluctuation and inter-specimen variability of Chl *a* content were larger in SWf than those in NPf. In contrast, the specimens in the unfed groups showed an overall decrease or no change in Chl *a* content, except over the first few days of the experiment (Fig. 4 b2, d2). The maximum Chl *a* contents were 79 ng foraminifer^−1^ in SW (sac59, day 4, 357 μm) and 48 ng foraminifer^−1^ in NP (sac67, day 4, 268 μm), and both were recorded at an early stage in the culture experiment.

### Photophysiological states

**Fig. 5:**
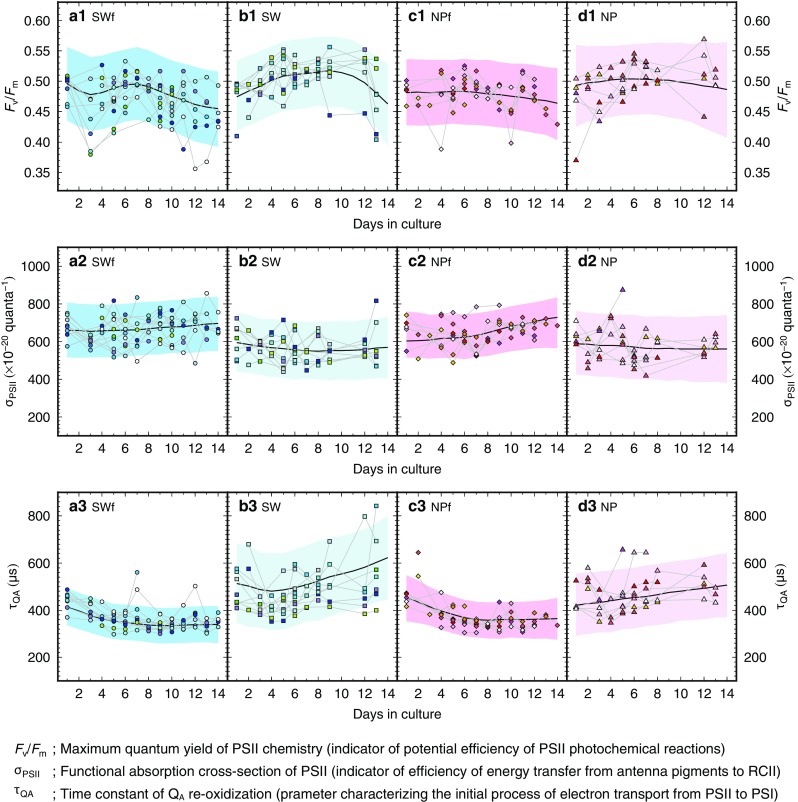
Longitudinal changes in the photophysiological parameters. (a1 to d1) *F*_v_/*F*_m_, (a2 to d2) σ_PSII_, and (a3 to d3) τ_QA_. **a** Group SWf, **b** Group SW, **c** Group NPf, and **d** Group NP. Bold lines represent the medians, and shaded areas represent the 95% intervals of the Bayesian posterior predictive distribution. PSII; photosystem II, RCII; reaction center of photosystem II. Please see the legend of Fig. 4 for the description of the symbols

Although the longitudinal trajectory of the photophysiological parameters (*F*_v_/*F*_m_, σ_PSII_, and τ_QA_) varied substantially with the individual, even in the same experimental group, the statistical model identified an overall trend for each group (Fig. 5). In the fed groups SWf and NPf, the median of the Bayesian posterior predictive distribution of the *F*_v_/*F*_m_ values decreased slightly through the culture period, and that of the σ_PSII_ values increased slightly in contrast (Fig. 5a1–a2, c1–c2). The predictive median of the τ_QA_ values decreased in the first ca. 6 days, but remained constant thereafter (Fig. 5a3, c3). On the other hand, in the unfed groups SW and NP, the predictive median of the *F*_v_/*F*_m_ and σ_PSII_ values showed no clear trend, while that of the τ_QA_ values increased (Fig. 5b1–b3, d1–d3).

The predictive median varied in the range of 0.45–0.52 for *F*_v_/*F*_m_, and 549–729 × 10^−20^ quanta^−1^ for σ_PSII_. The predictive median for τ_QA_ was in a range of 421–622 μs in the unfed groups, although in the fed groups, it had more constrained values within 334–452 μs. The minimum and the maximum values of the predictive median in the longitudinal profile did not deviate from the lower and upper ends of the 95% predictive interval, respectively, for the *F*_v_/*F*_m_ and σ_PSII_ in all groups (Fig. 5 a1–d1, a2–d2). For the predictive median for τ_QA_ in the fed groups, the maximum values observed on day 1 exceeded the upper end of the 95% interval after day 6 in SWf and after day 9 in NPf (Fig. 5 a3, c3). It can be concluded that τ_QA_ in the fed groups alone decreased significantly during the experiment.

**Fig. 6:**
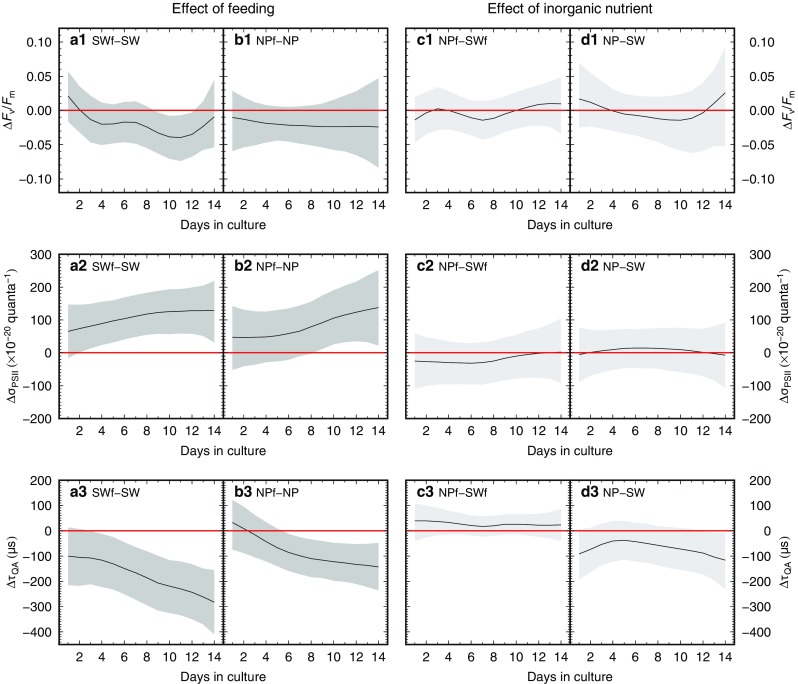
Differences in photophysiological parameters due to experimental treatments. (a1 to d1) difference in *F*_v_/*F*_m_, (a2 to d2) difference in σ_PSII_, (a3 to d3) difference in τ_QA_. **a** Groups SWf versus SW, **b** Groups NPf versus NP, **c** Groups NPf versus SWf, **d** Groups NP versus SW. Bold black lines represent the medians of the differences and the shaded areas represent and the 95% intervals of the Bayesian posterior predictive distribution. When the shaded area contained 0 (red line), the difference between two groups was statistically insignificant at that point

Comparing the fed and unfed groups under each nutritional condition, the difference in *F*_v_/*F*_m_ was not significant except for days 9–12 in the low-nutrient groups (SWf and SW) (Fig. 6 a1, b1). The *F*_v_/*F*_m_ tended to be lower in the fed groups. Δσ_PSII_ increased greatly as the day went on (Fig. 6a2, b2), whereas Δτ_QA_ decreased markedly (Fig. 6a3, b3). The differences in σ_PSII_ and τ_QA_ caused by feeding were profound under the low-nutrient condition. In contrast, the differences due to inorganic nutrient concentration were mostly insignificant throughout the period (Fig. 6c1–c3, d1–d3). Under starving condition alone, τ_QA_ showed a relatively large difference between the high- and low-nutrient groups (Fig. 6 d3), the values being low in the high-nutrient group NP.

### Photophysiological states of free-living *Pelagodinium béii* in culture

The photophysiological parameters of the dinoflagellate (*Pelagodinium béii*) when free-living in nutrient-replete media were comparable with those of the symbionts within the host (Table 3). The nutrient-replete culture yielded an *F*_v_/*F*_m_ value of ca. 0.5, σ_PSII_ value of ca. 600 × 10^−20^ quanta^−1^, and τ_QA_ value of ca. 500 μs, all of which were within the observed ranges of those in the host. There appeared to be no differences in photophysiological parameters between the two studied growth phases of *P. béii* in culture.

**Table 3:** Values of photophysiological parameters for free-living symbionts. The error (1σ) represents the analytical error of repeated measurements for each sample (*n* = 50). Note that the samples of different subcultures at different growth phases. (1) and (2) are 7 days and 10 days after each subculture, respectively. *F*_v_/*F*_m_; maximum quantum yield of photosystem II chemistry, σ_PSII_; functional absorption cross-section of photosystem II, τ_QA_; time constant of initial electron acceptor Q_A_ re-oxidization

	*F*_v_/*F*_m_	σ_PSII_ (× 10^−20^ quanta^−1^)	τ_QA_ (μs)	Growth phase
*Pelagodinium béii* (1)	0.500 ± 0.010	608 ± 20	515 ± 74	Exponential phase
*Pelagodinium béii* (2)	0.486 ± 0.012	586 ± 22	473 ± 76	Stationary phase

## Discussion

### Effect of feeding

The growth patterns and the Chl *a* content of the holobionts were clearly influenced by the feeding regime. Larger final test size, more chambers, and a higher ratio of gametogenesis were attained in the fed groups, demonstrating that foraminifers require prey to grow and achieve reproductive maturation (Fig. 3). In contrast, in the unfed groups, most of the specimens did not grow. These growth results were in agreement with previous findings by Bé et al. (1981). They examined *G. sacculifer* under several feeding regimes and demonstrated the necessity of food for foraminifer growth. In our study, we also showed that an increase in Chl *a* content was evident in the growing, fed foraminifers, whereas there was no change in the non-growing, unfed foraminifers. The dynamics of the Chl *a* content in the holobionts, reflecting the growth of the symbionts, were depicted quantitatively. Some specimens in the unfed groups which formed new chamber(s) until day 5, were probably fueled via the digestion of prey remnants that the foraminifers had fed on in their natural environment before collection. Since freshly collected planktic foraminifers usually have food remains in their cytoplasm (Anderson and Bé 1976; Anderson et al. 1979), being nourished for several days by these substances is plausible. Interestingly, the increase in Chl *a* content, reflecting an increase in the number of symbionts, continued until day 5, followed by decrease thereafter (Fig. 4). Metabolic waste from the hosts was likely to have kept the symbionts in a vigorous state during the earlier period of the experiment (until day 5 in this study). It possibly represents the duration time for the exhaustion of stored energy used by foraminifers for growth.

The cytoplasm reduction observed in the unfed groups could be a consequence of host starvation. The starving foraminifers may have digested their own cellular components. In the non-growing, starving host foraminifers, it is assumed that symbiont population density became reduced because of digestion of the symbionts or a shortage of metabolite supplied by the host. As a result of cytoplasm reduction, the test was observed to become progressively empty from the last-formed chamber (Fig. 2). We assumed that the spontaneous loss of the emptied chamber(s) may be a response to avoid sinking. The tests are made of CaCO_3_, and therefore, the density of empty *G. sacculifer* tests is ca. 2.7 g cm^−3^. Indeed, the weight of a chamber added to the pre-formed test of *G. sacculifer* has been reported to account for half of its total test weight (Takagi et al. 2015). Therefore, retaining the heavy, yet empty chamber would facilitate sinking. In the natural environment, sinking results in a decrease in the amount of light received and less opportunity of capturing prey in the water column. This would be disadvantageous for photosymbiotic foraminifers. In summary, the observed phenomena and behavior of the unfed specimens appeared to be a positive response for survival.

There were clear contrasts in the photophysiological parameters between the fed and unfed groups (Fig. 6). Relatively low *F*_v_/*F*_m_ and high σ_PSII_ were observed in the fed groups (Fig. 6a1–a2, b1–b2). Generally, this combination can be interpreted as a limitation of nutrient supply under an unbalanced growth condition (Kolber et al. 1988; Suggett et al. 2009). A decrease in *F*_v_/*F*_m_ corresponding to a nutrient limitation, indicates a reduction in the proportion of functional reaction centers of PSII. When the functional and the damaged reaction centers share a common light-harvesting antenna, it is accompanied by a relative increase in the functional cross-section of PSII (σ_PSII_) as a consequence (Falkowski and Kolber 1995; Suggett et al. 2004). In this respect, the fed groups showing a slight decrease in *F*_v_/*F*_m_ with high σ_PSII_ may have suffered a slight decrease in nutrient supply. This can be explained by considering the experimental condition and their growth profiles. The feeding regime of one *Artemia* every two days was not altered throughout the culture period (constant input of organic nutrition), even though the host size and the number of symbionts increased significantly with time, so that there was a growing demand for nutrients. This would cause a decrease in the quantity of available nutrients per algal cell. The Δ*F*_v_/*F*_m_ and Δσ_PSII_ decreased and increased, respectively, with the growth of the foraminifers (Fig. 6 a1–a2, b1–b2). Such correspondence between the decrease in *F*_v_/*F*_m_ and the increase in σ_PSII_ with an increase in Chl *a* content per foraminifer, was similar to that observed in the cultured planktic foraminifer *Globigerinella siphonifera* Type II (Fujiki et al. 2014). Furthermore, the τ_QA_ did not increase in the fed groups (Fig. 5a3, c3), indicating that a decrease in nutrient quantity, if any, did not damage successive electron transport for carbon fixation.

The elevated *F*_v_/*F*_m_ and lowered σ_PSII_ values observed in the unfed groups may indicate that the symbionts benefited from a better nutrient condition. However, it is unlikely that the symbionts of starving hosts, especially in the group SW with no apparent external nutrient source, would be able to photosynthesize under a better nutrient condition than in the fed groups. The observed cell-volume decrease itself indicates that the host was starving and not in a healthy condition. We also considered another possible scenario for the elevated *F*_v_/*F*_m_ and the decreased σ_PSII_ values in the symbionts of starving hosts. While the τ_QA_ was significantly higher in the unfed groups, it was noteworthy that the values remained within the usual range (~600 μs, Kolber and Falkowski 1993). A high τ_QA_ represents slow electron transport from the primary electron acceptor of PSII (Q_A_) to its downstream. In a situation of reduced electron transport capacity, light absorption should become excessive, consequently generating harmful hydrogen peroxide (Gorbunov et al. 2001; Smith et al. 2005), unless the size of the light-harvesting antenna is altered. A reduction in antenna size (σ_PSII_ downregulation) should occur for optimizing the light-harvesting system to balance energy in general (e.g. Norman et al. 1998). This would account for the observed low σ_PSII_ values accompanied by high τ_QA_ in the unfed groups. As such, if σ_PSII_ downregulation occurred in a manner independent of the number of functional reaction centers of PSII, it could theoretically cause an elevation in *F*_v_/*F*_m_, owing to the reverse mechanism for low *F*_v_/*F*_m_ and high σ_PSII_ (Falkowski and Kolber 1995) observed in the fed groups. This second possibility of antenna size reduction might be a more plausible explanation for the unfed groups. Simultaneously, for the fed groups, apart from the first assumption regarding the decrease in nutrient quantity in the cytoplasm, the high σ_PSII_ and low *F*_v_/*F*_m_ can also be achieved by changing antenna size. Since there were sufficient substances to synthesize accessory photopigments for the symbionts in the fed groups, the antenna system might have developed better, which could have caused σ_PSII_ elevation. Again, if the number of the reaction centers was unchanged, or its increase was smaller than that of the antenna photopigments, a decrease in *F*_v_/*F*_m_ can occur. Considering in a comprehensive manner, the change in antenna size appears to explain the response of both the fed and unfed groups consistently, although we require further research.

One notable aspect was that the photophysiological parameters of the nutrient-replete cultures of free-living *P. béii* were comparable with the parameters of those in the host. It suggested that the PSII of the symbionts in the foraminifers was not damaged severely, regardless of the seriously depleted nutritional conditions in this study. One of our most important findings was that unfed, starving holobionts remained photosynthetically competent for at least two weeks. However, the active fluorometry-based assessment of the net fitness of the holobionts requires further verification by analyzing isolated symbiotic algal cultures under various conditions. Once this is accomplished, active fluorometry will become a highly robust tool for understanding host-symbiont interactions.

### Apparent ineffectiveness of inorganic nutrients

We observed that the elevated nutrient concentration did not cause any significant difference in the growth of the foraminifers or symbionts (Fig. 4), and did not affect the photophysiological parameters (Fig. 6). These findings demonstrated that the symbionts in the host did not benefit from the inorganic nutrients in seawater even under the high-nutrient condition, regardless of the predation history of their host. It is well-known that the other photosymbiotic organisms, such as benthic foraminifers and corals respond to elevated nutrient levels positively or negatively (Lee et al. 1991; Hallock 2001; Tanaka et al. 2014; Rosset et al. 2015). Therefore, our results were differed from the expected response based on current knowledge of photosymbiotic consortia.

Our studies on the unfed holobionts indicated that their metabolism was fine, however, their growth was limited. The fact that even the symbionts in the SW group could maintain their photophysiology indicated that metabolic waste was supplied to the symbionts via the basal metabolism of the host. Although it led to the destruction of the cytoplasm of the host to a certain extent, it was not fatal for at least 12 days. We assumed that this minimal nutrient supply was sufficient for the symbionts to maintain their photosynthetic fitness, and that this was responsible for the ineffectiveness of additional inorganic nutrients on the photophysiology. An alternate possibility that cannot be excluded, is a mutualistic association with diazotrophic bacteria. However, no prokaryotic symbiosis is known for this taxon but if nitrogen-fixing organisms were present in the cytoplasm of the host, they could mediate photosynthesis by providing a nitrogen supply (Lema et al. 2012).

The simplest explanation to account for the non-growing nature of the holobionts in group NP, was that they were incapable of incorporating inorganic nutrients outside their membranes. However, this appears unrealistic. Symbionts within the host are enveloped by a membrane of the host within the cytoplasm. A transport system should therefore exist, for exchange of materials, at the membrane separating host cytoplasm from that of the symbionts (the symbiosome membrane). The symbiosome is formed via endocytosis of a symbiont cell; therefore, its membrane is identical to that separating the host from the surrounding seawater. Thus, it is reasonable to consider the function of the two membranes to be the same.

The other possibility is that the holobionts in the NP group could incorporate and utilize the nutrients in seawater but their growth was limited by unknown factors. This might involve another nutrient such as iron that is widely known to limit phytoplankton growth (e.g. Kolber et al. 1994). However, lack of growth might be due to a strict control exerted by the host foraminifers that regulates the number of symbionts. We know that planktic foraminifers completely control the deployment or withdrawal of their symbionts in response to the light condition (Bé et al. 1977). It would therefore not be surprising if the hosts regulate the growth of the symbionts as well so that the hosts allow the symbionts to live well enough to photosynthesize, but do not allow them to grow. Such control is observed in photosymbiotic relationships of corals (Falkowski et al. 1993). However, the difference is that corals do respond to elevated inorganic nutrient levels unlike planktic foraminifers. If the limitation of symbiont growth is induced by the host, it implies that planktic foraminifer hosts can exercise stricter control on their symbionts than coral hosts perhaps by regulating accessibility to nutrients in seawater. From a different viewpoint, it can also be considered as a mechanism for protecting the symbionts from the changing environmental conditions, thus, establishing a highly stable photosymbiosis.

### Other possibilities and implication for a function of photosymbiosis

The results of this study indicated that the contribution of the symbiont photosynthates to the nutrition of the host was significantly smaller than that of the *Artemia*-derived catabolites. In fact, there was no direct evidence for the contribution of photosynthates to the host. The symbionts within the starving host did not appear to utilize the inorganic nutrients present in seawater, unlike the well-known photosymbiotic relationship of corals and that of benthic foraminifers. However, if the external dissolved nitrogen is in the form of ammonium ion, and not nitrate, the results may be different. Uhle et al. (1999) proposed that nitrogen is efficiently recycled within the photosymbiotic systems via a recycled NH_4_^+^ pool, suggesting the importance of ammonium over the other forms of nitrogen. Whether the symbionts can proliferate using ammonium ions present in seawater is a subject for further research. We concluded that the advantage of photosymbiosis to planktic foraminifers, at least to *G. sacculifer*, is not daily nutrition, implying that there must be another function(s).

One straightforward explanation is that symbionts are useful as suppliers of “emergency food”. In this study, most of the starving hosts could survive for 12 days while retaining photosynthetic fitness. Thus, in spite of the consumption of their own cellular components, a feature of the starving specimens, photosynthesis may provide adequate nutrition to the host for its survival and nutrients recycled effectively between the two partners. Future studies using various light conditions and starvation should provide the answer to this question. The other possibility is that the symbionts are essential for the host reproduction. At the end of their life cycle, the rapid digestion or lysis of a large number of symbionts by the matured host was reported (Bé et al. 1983; Takagi et al. 2016). It is suggested that symbionts are a source of energy for reproduction (Bé et al. 1983). Alternatively, hosts may rely on their symbionts for metabolite processing such as the elimination of host metabolites such as ammonia. This may occur via active utilization through symbiont photosynthesis. Thus, symbionts could contribute by reducing the cost to the host of ammonia transport to the outside of the cell. Generally, in closely-related symbiotic consortia, in which there is a great dependence of the host for certain functions, its partner may lose their own related metabolic pathways (e.g. Shinzato et al. 2011). If the host foraminifers have become dependent enough on their symbionts for some of these life processes, such as metabolite elimination, and synthesis of essential amino acids or vitamins, the hosts must not lose their symbionts. These metabolic relationships have not previously been considered as an important function of the photosymbiosis in planktic foraminifers and would be an interesting target for future research. This would help us to understand the obligate interaction between planktic foraminifers and their symbionts.

## Electronic supplementary material

ESM 1 (PDF 757 kb)
