# Supplementary material for: Effect of nutritional condition on photosymbiotic consortium of cultured Globigerinoides sacculifer (Rhizaria, Foraminifera)
Source: Symbiosis. 2017 Dec 9;76(1):25–39. doi: 10.1007/s13199-017-0530-3 (PMC6096716; doi:10.1007/s13199-017-0530-3)
Supplement: Supplementary file 1 — (PDF 757 kb) [file 13199_2017_530_MOESM1_ESM.pdf]

Electronic Supplementary Material
Journal: *Symbiosis*

Effect of nutritional condition on photosymbiotic consortium of cultured *Globigerinoides sacculifer* (Rhizaria, Foraminifera)

Haruka Takagi · Katsunori Kimoto · Tetsuichi Fujiki · Kazuyoshi Moriya

H. Takagi (✉) *
Graduate School of Creative Science and Engineering, Waseda University,
1-6-1 Nishiwaseda, Shinjuku, Tokyo 169-8050, Japan
*Current address:
Atmosphere and Ocean Research Institute, The University of Tokyo
5-1-5 Kashiwanoha, Kashiwa, Chiba 277-8564, Japan
htakagi@aori.u-tokyo.ac.jp
harurah-t@fuji.waseda.jp
+81-4-7136-6367

K. Kimoto, T. Fujiki
Research and Development Center for Global Change, Japan Agency for Marine-Earth Science and Technology,
2-15 Natsushima-cho, Yokosuka, Kanagawa 237-0061, Japan

K. Moriya
Department of Earth Sciences, Faculty of Education and Integrated Arts and Sciences, Waseda University,
1-6-1 Nishiwaseda, Shinjuku, Tokyo 169-8050, Japan

Table S1 Results of FRR fluorometric measurement for group SWf.

ID	Day	Size (μm)	F_0 (dimensionless)	F_m (dimensionless)	F_v/F_m (dimensionless)	σ_{PSII} ($\times 10^{-20}$ quanta^{-1})	τ_{Qa} (μs)
Initial test size < 400 μm							
sac17	1	321	0.8	1.7	0.506	657	411
	3	321	2.1	3.8	0.438	553	378
	5	643	2.2	4.1	0.467	614	358
	6	732	2.2	4.0	0.448	643	376
	7	732	0.2	0.4	0.494	834	561
sac18	1	250	1.9	3.7	0.488	715	370
	3	250	1.6	3.2	0.504	611	358
	5	464	1.8	3.7	0.506	560	360
	6	464	1.2	2.4	0.491	524	396
	7	464	2.4	4.8	0.505	577	367
	8	679	4.5	9.0	0.505	648	317
	9	679	4.9	9.6	0.494	667	318
	10	679	4.8	8.7	0.451	580	362
	11	679	3.8	7.4	0.493	620	332
	12	679	2.7	4.9	0.454	484	360
	13	768	3.6	6.8	0.465	674	348
	14	768	3.8	6.9	0.448	643	359
sac31	1	339	0.7	1.5	0.509	682	438
	3	482	1.9	3.0	0.380	597	432
	5	607	2.3	3.9	0.422	626	352
	6	607	3.0	5.5	0.458	576	346
	7	607	NA	NA	NA	NA	NA
	8	786	3.1	6.1	0.489	714	325
	9	786	3.4	6.0	0.435	696	385
	10	786	4.0	7.8	0.490	699	325
sac32	1	339	0.6	1.2	0.504	685	412
	3	339	1.7	3.3	0.471	605	373
	5	500	1.4	2.7	0.498	542	380
	6	500	2.8	5.6	0.499	584	352
	7	500	NA	NA	NA	NA	NA
	8	714	4.6	9.0	0.494	602	311
	9	714	4.1	8.4	0.517	611	301
	10	714	6.1	10.8	0.432	569	344
	11	714	3.9	8.1	0.517	594	318
	12	714	5.7	11.1	0.484	711	334
	13	750	4.0	7.5	0.467	609	346
sac33	1	286	0.5	1.0	0.500	751	466
	3	375	0.9	1.6	0.470	628	426
sac39	1	304	0.4	0.7	0.488	678	455
	3	393	1.6	2.8	0.414	580	392
	5	518	NA	NA	NA	NA	NA
	6	518	4.4	8.7	0.495	742	344
	7	518	NA	NA	NA	NA	NA
	8	696	3.8	7.3	0.484	708	330
	9	696	3.5	6.8	0.492	766	314
	10	696	3.2	6.2	0.485	693	334

ID	Day	Size (μm)	F_0 (dimensionless)	F_m (dimensionless)	F_v/F_m (dimensionless)	σ_{PSII} ($\times 10^{-20}$ quanta^{-1})	τ_{Qa} (μs)
Continued							
sac42	1	339	0.9	1.7	0.462	741	441
	3	339	1.3	2.2	0.385	635	450
	5	500	3.1	5.3	0.415	719	387
	6	500	NA	NA	NA	NA	NA
	7	661	NA	NA	NA	NA	NA
	8	661	3.8	6.8	0.437	737	356
	9	661	5.0	9.2	0.457	777	343
	10	804	3.4	6.1	0.444	659	364
	11	804	4.5	8.5	0.469	710	355
	12	804	3.3	5.9	0.434	716	388
sac51	1	321	0.4	0.8	0.458	574	462
	4	321	1.9	3.5	0.463	645	374
	5	429	1.1	2.1	0.469	517	366
	6	429	NA	NA	NA	NA	NA
	7	429	1.1	2.2	0.495	574	403
	8	554	NA	NA	NA	NA	NA
	9	554	5.9	11.1	0.473	733	323
	10	554	6.6	12.2	0.458	724	332
	11	554	6.2	11.9	0.479	709	344
	12	804	5.1	9.3	0.454	705	385
	13	804	5.2	9.3	0.442	668	309
	14	804	3.7	6.6	0.434	651	390
sac52	1	250	0.3	0.5	0.502	637	487
	4	250	1.4	3.0	0.527	683	362
	5	375	3.2	5.8	0.451	817	355
	6	375	3.0	6.3	0.517	664	337
	7	375	2.3	4.6	0.506	607	359
	8	554	NA	NA	NA	NA	NA
	9	554	4.6	8.2	0.442	748	340
	10	554	8.7	15.3	0.432	766	307
	11	554	6.7	11.0	0.388	684	357
	12	732	5.1	9.0	0.425	744	380
	13	732	4.5	7.8	0.427	674	332
	14	732	4.1	7.3	0.435	666	350
sac61	2	321	NA	NA	NA	NA	NA
	4	321	3.3	6.5	0.493	789	334
	5	482	6.7	13.1	0.490	746	298
	6	482	5.2	11.1	0.533	666	303
	7	482	4.6	9.6	0.517	695	316
	8	732	NA	NA	NA	NA	NA
	9	732	4.0	7.8	0.487	693	325
	10	732	6.4	11.9	0.464	723	323
	11	732	4.1	8.2	0.498	810	319
	12	732	4.2	8.3	0.500	747	327
	13	982	5.1	10.3	0.507	747	302
	14	982	3.4	6.8	0.493	652	329

ID	Day	Size (μm)	F_0 (dimensionless)	F_m (dimensionless)	F_v/F_m (dimensionless)	σ_{PSII} ($\times 10^{-20}$ quanta^{-1})	τ_{Qa} (μs)
Continued							
sac62	2	250	NA	NA	NA	NA	NA
	4	286	1.4	2.9	0.506	724	334
	5	500	4.7	8.9	0.471	665	324
	6	500	6.0	12.2	0.510	711	320
	7	500	4.3	8.9	0.516	663	348
	8	607	NA	NA	NA	NA	NA
	9	607	3.2	6.0	0.464	609	348
	10	607	2.7	5.1	0.479	632	363
sac65	2	250	NA	NA	NA	NA	NA
	4	250	0.5	1.0	0.455	666	436
	5	250	1.2	2.3	0.473	716	394
	6	250	1.2	2.3	0.473	618	388
	7	339	0.3	0.5	0.474	495	487
	8	339	NA	NA	NA	NA	NA
	9	339	1.4	2.4	0.426	587	383
	10	446	2.1	3.6	0.421	566	363
	11	446	0.9	1.7	0.442	540	421
	12	446	1.5	2.3	0.356	659	503
	13	661	3.9	6.2	0.368	855	366
	14	661	3.2	5.5	0.425	756	361
Initial test size > 400 μm							
sac4	1	500	3.5	7.0	0.507	640	332
	3	500	1.7	3.2	0.457	513	375
sac6	1	589	1.9	3.7	0.490	531	379
	3	589	3.0	5.0	0.410	589	366
sac8	1	589	1.3	2.4	0.460	641	429
	3	589	0.3	0.5	0.270	633	734
sac16	1	500	1.9	3.5	0.468	596	355
	3	571	3.0	5.8	0.483	641	338
sac46	1	518	1.8	3.6	0.490	704	398
sac55	1	607	0.6	1.1	0.450	578	551
	4	607	0.9	1.5	0.410	568	400

Table S2 Results of FRR fluorometric measurement for group SW.

ID	Day	Size (μm)	F_0 (dimensionless)	F_m (dimensionless)	F_v/F_m (dimensionless)	σ_{PSII} ($\times 10^{-20}$ quanta^{-1})	τ_{Qa} (μs)
Initial test size < 400 μm							
sac15	1	357	0.4	0.8	0.482	665	435
	3	357	0.7	1.5	0.521	603	392
	5	357	0.2	0.5	0.552	509	465
	6	357	0.2	0.4	0.511	661	473
	7	357	NA	NA	NA	NA	NA
	8	357	NA	NA	NA	NA	NA
sac25	1	357	0.7	1.3	0.496	671	430
	3	357	0.8	1.5	0.481	546	457
	5	357	0.6	1.2	0.459	440	568
	6	214	0.4	0.7	0.493	568	574
	7	214	NA	NA	NA	NA	NA
	8	214	0.2	0.5	0.504	661	496
	9	143	0.1	0.3	0.508	586	692
	12	143	NA	NA	NA	NA	NA
sac27	1	321	0.5	0.9	0.486	618	435
	3	321	0.5	0.9	0.495	495	425
	5	321	0.4	0.9	0.541	461	442
	6	321	0.5	1.1	0.527	553	433
	7	321	NA	NA	NA	NA	NA
	8	321	NA	NA	NA	NA	NA
	9	321	NA	NA	NA	NA	NA
	9	214	0.2	0.4	0.537	518	571
sac30	1	375	0.8	1.6	0.495	723	409
	3	375	1.2	2.2	0.478	587	381
	5	375	0.7	1.4	0.518	494	404
	6	375	0.7	1.4	0.509	502	426
	7	375	NA	NA	NA	NA	NA
	8	375	0.7	1.5	0.535	630	441
	9	375	NA	NA	NA	NA	NA
	12	375	0.4	0.9	0.552	555	419
	13	375	0.4	0.7	0.479	523	488
sac35	1	250	0.3	0.6	0.494	670	532
	3	250	0.7	1.4	0.501	600	402
	5	250	0.2	0.4	0.549	456	447
	6	250	0.1	0.3	0.528	562	491
	8	250	0.1	0.2	0.537	669	570
	9	179	NA	NA	NA	NA	NA
	12	179	NA	NA	NA	NA	NA
sac49	1	375	0.5	0.9	0.410	497	466
	4	375	NA	NA	NA	NA	NA
	5	375	NA	NA	NA	NA	NA

ID	Day	Size (μm)	F_0 (dimensionless)	F_m (dimensionless)	F_v/F_m (dimensionless)	σ_{PSII} ($\times 10^{-20}$ quanta^{-1})	τ_{Qa} (μs)
Continued							
sac50	1	214	0.2	0.5	0.494	560	563
	4	214	0.8	1.7	0.530	595	400
	5	286	0.7	1.5	0.518	609	387
	6	286	0.6	1.1	0.496	536	456
	7	286	0.4	0.8	0.518	488	462
	8	286	0.3	0.6	0.520	475	538
	9	286	0.3	0.6	0.522	555	594
	12	286	0.4	0.9	0.531	587	493
	13	286	0.3	0.5	0.478	471	541
sac57	1	232	0.1	0.3	0.494	503	576
	4	232	1.2	2.3	0.474	637	462
	5	232	0.8	1.7	0.537	621	385
	6	232	0.3	0.5	0.490	486	594
	7	232	0.3	0.6	0.499	495	551
	8	232	0.2	0.5	0.529	495	508
	9	232	0.3	0.8	0.556	493	505
	12	232	0.4	0.8	0.533	533	476
	13	232	0.2	0.4	0.404	469	843
sac59	2	339	0.2	0.4	0.469	561	575
	4	357	2.2	4.4	0.505	649	352
	5	357	2.0	4.0	0.486	715	354
	6	357	0.9	1.8	0.504	570	398
	7	357	0.7	1.4	0.517	447	410
	8	357	0.8	1.6	0.511	496	468
	9	357	1.0	1.8	0.444	549	436
	12	357	0.9	1.7	0.447	610	450
	13	357	0.9	1.6	0.413	817	469
sac63	2	268	0.1	0.3	0.440	476	680
	4	250	0.7	1.4	0.513	524	417
	5	250	0.8	1.6	0.513	603	397
	6	250	0.5	1.0	0.543	499	398
	7	250	0.3	0.6	0.494	553	503
	8	250	0.2	0.5	0.510	654	477
	9	250	0.3	0.6	0.532	503	497
	12	250	0.1	0.2	0.515	518	798
	13	196	0.2	0.3	0.453	701	693
sac70	2	214	1.2	2.5	0.495	658	401
	4	321	0.9	2.0	0.539	539	367
	5	321	1.4	2.7	0.480	602	452
	6	321	1.2	2.5	0.523	685	377
	7	321	0.6	1.2	0.520	544	413
	8	321	NA	NA	NA	NA	NA
	9	321	0.9	1.8	0.515	490	414
	12	321	0.8	1.8	0.538	527	384
	13	321	0.9	1.9	0.521	549	400

ID	Day	Size (μm)	F_0 (dimensionless)	F_m (dimensionless)	F_v/F_m (dimensionless)	σ_{PSII} ($\times 10^{-20}$ quanta^{-1})	τ_{Qa} (μs)
Continued							
Initial test size > 400 μm							
sac1	1	464	2.1	4.3	0.500	580	420
	3	464	0.9	1.5	0.420	455	458
	4	464	1.0	1.7	0.420	533	454
	5	464	0.3	0.5	0.470	759	596
	6	464	NA	NA	NA	NA	NA
sac2	1	446	1.8	3.5	0.480	593	421
	3	589	2.2	4.2	0.480	558	364
	4	589	1.4	2.5	0.450	550	402
	5	589	1.5	2.9	0.470	621	377
	6	589	1.6	3.2	0.490	658	368
	7	589	1.2	2.2	0.480	565	405
	8	589	1.4	2.8	0.480	690	399
	9	589	0.3	0.4	0.360	732	NA
sac3	1	429	2.3	4.9	0.520	615	383
	3	429	0.8	1.3	0.410	533	498
	4	429	0.2	0.4	0.420	709	515
	5	429	NA	NA	NA	NA	NA
sac13	1	464	1.9	3.7	0.482	575	403
	3	464	1.0	1.9	0.490	521	380
	4	464	NA	NA	NA	NA	NA
	5	464	0.8	1.6	0.505	531	408
	6	464	0.6	1.3	0.517	523	395
	7	464	0.6	1.3	0.520	534	393
	8	464	0.5	1.0	0.537	542	424
	9	464	0.5	1.1	0.509	510	430
	12	464	0.4	0.8	0.511	551	453
	13	464	0.5	1.0	0.459	625	469
sac14	1	446	1.1	2.3	0.502	725	357
	3	446	1.6	3.4	0.522	590	336
	4	446	NA	NA	NA	NA	NA
	5	446	0.8	1.7	0.518	661	395
	6	446	0.8	1.7	0.516	666	410
	7	446	0.6	1.3	0.535	565	415
	8	446	0.8	1.7	0.540	660	388
	9	446	0.4	0.9	0.522	593	469
	12	304	0.4	0.8	0.554	628	420
	13	304	0.3	0.5	0.495	539	540
sac26	1	411	0.8	1.5	0.490	646	420
	3	411	1.4	2.7	0.501	595	369
	5	411	0.7	1.4	0.510	503	393
	6	411	0.5	1.1	0.501	568	466
	7	411	NA	NA	NA	NA	NA
	8	411	0.4	0.9	0.528	685	427
	9	286	NA	NA	NA	NA	NA
	13	286	NA	NA	NA	NA	NA
sac28	1	714	2.1	4.2	0.500	633	392
	3	714	2.0	3.7	0.470	584	528

Table S3 Results of FRR fluorometric measurement for group NPf.

ID	Day	Size (μm)	F_0 (dimensionless)	F_m (dimensionless)	F_v/F_m (dimensionless)	σ_{PSII} ($\times 10^{-20}$ quanta^{-1})	τ_{Qa} (μs)
Initial test size < 400 μm							
sac22	1	393	1.1	2.0	0.459	740	415
	3	393	1.4	2.6	0.460	632	382
	4	536	NA	NA	NA	NA	NA
	5	536	1.2	2.2	0.470	488	422
	6	625	1.1	2.1	0.461	595	417
sac34	1	339	0.7	1.4	0.501	549	453
	5	446	1.9	3.9	0.504	632	363
	6	446	2.7	5.6	0.525	654	345
	7	625	2.1	4.1	0.487	523	338
	8	625	3.3	6.4	0.489	616	326
	9	625	3.4	6.1	0.453	591	434
	10	804	3.3	6.0	0.450	689	352
	11	804	4.8	9.9	0.517	643	307
	12	804	3.1	6.1	0.491	630	339
sac43	1	321	0.3	0.6	0.487	666	473
	3	321	NA	NA	NA	NA	NA
	5	464	NA	NA	NA	NA	NA
	6	464	2.3	4.7	0.521	628	340
	7	464	1.7	3.4	0.496	525	355
	8	679	3.1	6.0	0.483	606	330
	9	679	3.8	7.6	0.494	713	307
	10	679	4.1	6.8	0.398	668	429
	11	679	2.9	5.9	0.513	684	328
	12	679	3.0	6.0	0.503	608	337
sac44	1	357	0.7	1.3	0.485	697	468
	3	357	NA	NA	NA	NA	NA
	5	500	2.0	3.8	0.479	644	348
	6	500	2.5	4.9	0.488	603	355
	7	732	2.4	4.5	0.475	526	344
	8	732	3.1	5.9	0.471	599	336
	9	786	3.2	6.4	0.495	654	331
sac48	1	393	0.7	1.3	0.494	678	437
	4	393	0.8	1.2	0.388	578	475
	5	393	1.4	2.8	0.489	613	362
	7	589	1.6	3.0	0.475	534	363
	8	589	3.8	7.1	0.471	708	343
	9	750	4.4	8.7	0.492	691	317
	10	750	4.1	8.1	0.490	675	330
	11	750	6.8	13.4	0.490	693	316
	12	750	5.8	11.0	0.470	702	332
sac58	2	268	0.3	0.5	0.472	508	544
	4	268	1.5	2.8	0.479	663	385

ID	Day	Size (μm)	F_0 (dimensionless)	F_m (dimensionless)	F_v/F_m (dimensionless)	σ_{PSII} ($\times 10^{-20}$ quanta^{-1})	τ_{Qa} (μs)
Continued							
sac60	2	304	NA	NA	NA	NA	NA
	4	464	5.5	10.5	0.478	745	326
	5	464	4.4	8.4	0.473	754	330
	6	571	5.0	10.1	0.509	732	305
	7	571	6.3	12.5	0.499	784	305
	8	571	NA	NA	NA	NA	NA
	9	821	2.4	4.5	0.465	793	368
sac68	2	286	NA	NA	NA	NA	NA
	4	286	1.4	2.9	0.518	736	368
	5	375	1.8	3.4	0.482	693	358
	6	375	1.8	3.7	0.496	654	354
	7	375	2.4	4.9	0.517	577	332
	8	375	NA	NA	NA	NA	NA
	9	571	NA	NA	NA	NA	NA
	10	571	2.6	4.7	0.447	620	393
	11	571	4.0	7.7	0.485	728	333
	12	571	3.2	6.1	0.478	671	352
	13	893	6.1	11.1	0.445	721	380
	14	893	8.1	14.2	0.429	684	336
sac71	2	357	0.2	0.4	0.460	637	645
sac72	2	286	NA	NA	NA	NA	NA
	4	286	1.1	2.3	0.513	568	381
	5	446	1.3	2.3	0.448	530	414
	6	446	2.2	4.4	0.503	641	361
	7	446	3.0	5.9	0.491	623	356
	8	446	NA	NA	NA	NA	NA
	9	625	4.7	8.8	0.465	703	351
	10	625	4.1	7.5	0.453	693	366
	11	625	3.9	7.7	0.489	655	352
	12	625	3.7	7.0	0.468	711	389
	13	625	4.0	7.3	0.455	693	374
Initial test size > 400 μm							
sac7	1	696	3.2	6.1	0.486	617	347
	3	696	3.3	5.9	0.443	588	374
sac11	1	411	2.0	4.0	0.510	692	378
	3	589	3.4	6.8	0.492	633	310
sac12	1	464	1.9	3.8	0.511	641	346
	3	643	2.3	4.0	0.420	655	376
sac23	1	536	1.8	3.6	0.504	645	380
	3	536	3.0	6.0	0.496	593	339
sac24	1	464	0.7	1.4	0.510	738	357
sac29	1	500	0.9	1.7	0.480	645	410
	3	500	0.5	0.8	0.410	574	515
sac45	1	536	1.2	2.5	0.500	658	401
sac56	1	482	0.4	0.8	0.490	502	445

Table S4 Results of FRR fluorometric measurement for group NP.

ID	Day	Size (μm)	F_0 (dimensionless)	F_m (dimensionless)	F_v/F_m (dimensionless)	σ_{PSII} ($\times 10^{-20}$ quanta^{-1})	τ_{Qa} (μs)
Initial test size < 400 μm							
sac20	1	375	0.9	1.7	0.489	629	414
	3	375	1.3	2.7	0.511	667	351
	4	375	NA	NA	NA	NA	NA
	5	375	0.5	0.9	0.480	594	417
	6	375	0.5	1.1	0.536	525	413
	7	375	0.6	1.3	0.531	624	413
	8	375	0.7	1.4	0.496	616	433
	9	375	NA	NA	NA	NA	NA
	12	286	0.4	0.8	0.504	569	512
	13	286	0.6	1.2	0.493	566	432
sac37	1	393	0.8	1.6	0.480	599	413
	3	393	0.9	1.6	0.434	655	443
	5	393	0.1	0.2	0.483	874	657
	6	393	NA	NA	NA	NA	NA
	7	393	NA	NA	NA	NA	NA
	8	393	NA	NA	NA	NA	NA
sac38	1	393	0.6	1.3	0.504	639	414
	3	393	0.5	1.1	0.523	505	382
	5	393	0.2	0.4	0.532	502	414
	6	393	0.2	0.4	0.529	552	450
	7	393	0.3	0.6	0.507	667	434
	8	393	0.3	0.6	0.501	592	568
	9	268	NA	NA	NA	NA	NA
	12	268	0.1	0.3	0.569	517	539
	13	268	0.2	0.4	0.506	594	495
sac40	1	339	0.5	0.8	0.370	585	525
	3	339	0.4	0.7	0.465	518	486
	5	339	0.4	0.7	0.466	583	461
	6	339	0.2	0.4	0.491	519	501
	7	339	0.2	0.3	0.496	519	518
	8	339	0.1	0.3	0.501	514	518
	9	161	NA	NA	NA	NA	NA
sac54	1	339	0.3	0.5	0.490	553	486
	4	339	0.4	0.8	0.474	531	493
	5	339	0.6	1.1	0.492	636	472
	6	339	0.5	0.9	0.478	565	494
	7	339	0.4	0.9	0.493	617	451
	8	339	NA	NA	NA	NA	NA
	9	339	NA	NA	NA	NA	NA
	12	339	0.1	0.2	0.428	492	713
	13	339	0.1	0.3	0.513	552	574
sac64	2	375	0.3	0.7	0.510	611	490
sac66	2	232	0.1	0.3	0.487	534	580
	4	232	0.5	0.9	0.475	637	448
	5	232	0.4	0.9	0.502	678	462
	6	232	0.1	0.3	0.537	498	644
	7	232	0.1	0.2	0.524	500	645
	8	170	NA	NA	NA	NA	NA
	9	170	NA	NA	NA	NA	NA
	10	170	NA	NA	NA	NA	NA

ID	Day	Size (μm)	F_0 (dimensionless)	F_m (dimensionless)	F_v/F_m (dimensionless)	σ_{PSII} ($\times 10^{-20}$ quanta^{-1})	τ_{Qa} (μs)
Continued							
sac67	2	268	0.2	0.4	0.490	489	518
	4	268	1.3	2.7	0.522	723	346
	5	268	0.6	1.4	0.532	583	386
	6	268	0.3	0.7	0.536	517	460
	7	268	0.2	0.5	0.532	418	492
	8	268	NA	NA	NA	NA	NA
	9	268	NA	NA	NA	NA	NA
	12	268	0.3	0.5	0.511	532	498
	13	268	0.2	0.5	0.519	641	467
sac69	2	286	0.2	0.3	0.504	458	535
	4	286	1.1	2.1	0.505	735	368
	5	286	0.7	1.3	0.509	629	393
	6	286	0.5	1.2	0.545	453	391
	9	214	NA	NA	NA	NA	NA
	12	125	0.2	0.3	0.441	540	592
Initial test size > 400 μm							
sac5	1	696	4.1	8.1	0.490	582	319
	3	696	3.0	5.4	0.440	674	485
sac9	1	554	2.1	4.1	0.490	691	370
sac10	1	571	0.6	1.1	0.470	671	450
sac19	1	554	2.2	4.4	0.493	612	384
	3	554	2.7	5.4	0.497	644	342
	4	554	NA	NA	NA	NA	NA
	5	554	NA	NA	NA	NA	NA
sac21	1	554	1.3	2.7	0.505	768	395
	3	554	1.4	2.9	0.503	568	358
	5	554	0.3	0.6	0.516	704	523
	6	554	NA	NA	NA	NA	NA
sac36	1	464	0.9	1.7	0.502	713	395
	3	464	0.8	1.5	0.442	581	432
sac41	1	400	0.6	1.1	0.468	710	406
	3	400	0.9	1.6	0.449	556	409
	5	400	0.3	0.7	0.487	485	472
	6	400	0.3	0.7	0.487	475	470
	7	400	0.4	0.9	0.523	515	439
	8	400	0.5	1.0	0.518	597	441
	9	400	NA	NA	NA	NA	NA
	12	400	0.6	1.4	0.542	609	446
	13	400	0.7	1.3	0.492	623	432
sac47	1	464	1.4	2.7	0.484	688	408
	3	536	1.3	2.4	0.463	552	397
	5	536	0.9	1.8	0.497	555	363
	6	536	0.9	1.8	0.499	559	400
	7	536	1.0	1.9	0.502	528	380
	8	536	1.6	3.3	0.517	636	377
	9	536	NA	NA	NA	NA	NA
	12	536	1.2	2.5	0.520	609	392
	13	536	1.0	1.9	0.491	580	403
sac53	1	518	0.4	0.8	0.490	488	489
	4	518	0.2	0.4	0.470	929	534

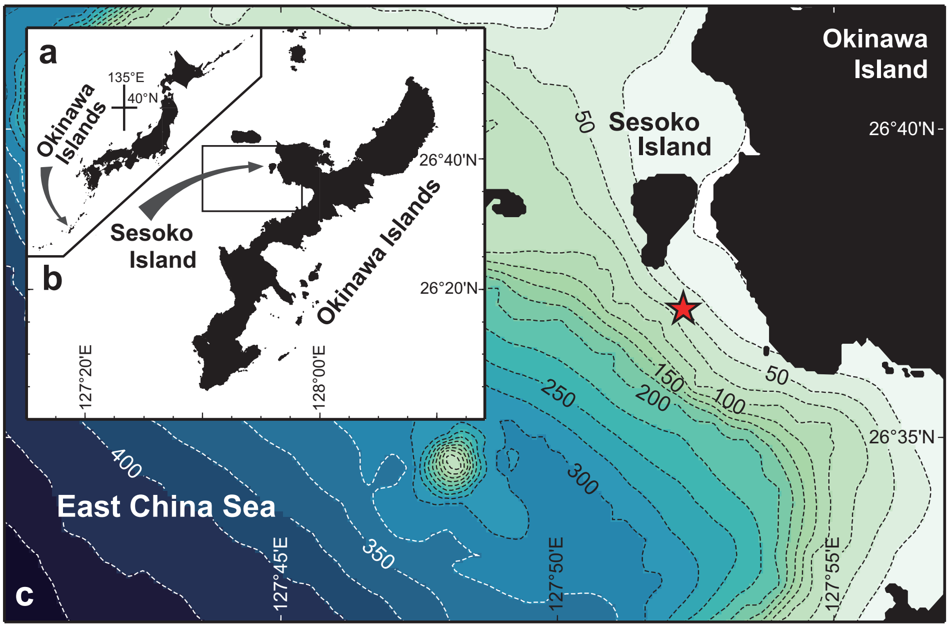

Fig. S1 Index map of the sampling site. (a) Map showing the location of the Okinawa Islands. (b) Map showing the location of the Sesoko Island. Inset box shows the area of (c). (c) Location of the sampling site (red star, 26°37.3'N, 127°52.3'E, 60-m deep).
